# Thoughts on General Education

**Published:** 1883-07

**Authors:** J. B. Chandler


### THOUGHTS ON GENERAL EDUCATION.

J. B. CHANDLER.

This is essentially an era of changes. Old landmarks, both physical and moral, are giving way before wants and ideas resulting from increasing knowledge and its more general diffusion.

What once were luxuries have become almost necessaries of life. The homes of some of the working people of our cities are superior in actual comforts to many of the palaces of the past. The people who live in these homes are no longer under the ban of ignorance. Education has made them capable to read and think for themselves, and reading and thinking are fast bearing them beyond the toils of superstition or of absurdities "which once held the world in awe."

"Truth is mighty and will prevail." Mere speculations as to the past or the future will no longer satisfy these inquirers of the nineteenth century. The chaff must be scattered before the whirlwind of investigation, and it is to be feared that in the crucial test of science, much, very much, that the weak and confiding have cherished as solemn and established truth upon which to base hope or fear, will be swept away as part of false teachings of designing men who built power for themselves upon the ignorance of their disciples.

Of course, truth will shine out only brighter for the removal of all this dross, but the transition from blind confidence in false teachings to the realization of that high standard of ability which unveils the wrong, will be attended by great danger.

The proof which establishes the fallacy of hopes and fears based upon weak and untenable doctrines which, from time to time, have been engrafted upon the various sectarian beliefs of the people, may not only tend to dispel those errors, but may, probably, with many, destroy entirely the confidence in all teachers and teachings, and thus delay the progress of truth.

This problem is being gradually worked out; indeed, it is working out itself, although many are watching with anxiety its course and the results.

There is nothing in the advanced state of thought, so far, which interferes in the least with the cherished hope of a large portion of the inhabitants of the world in a hereafter. Nor is there any step yet made in our advancing progress which disproves or proves any so-called revelation as to the future. Nor does it seem necessary to man's happiness here or hereafter that the question should be indisputably settled. Many find comfort in the affirmative, whilst not a few are not inconsolable in the negative.

Experience has proven, without power of dispute, that the very happiest way that men can live together on this earth is in the exercise of the great fundamental principles inculcated in the teachings of the Christian religion when divested of the dross above referred to.

Science has proven indisputably that the universe is governed by fixed and immutable laws.

Those principles and laws will continue through all convulsions which may arise in society in the struggle out of darkness into light. The only safeguard, however, in the transition is to be found in the earnest teaching and dissemination of those principles and laws untramelled by bigotry or worse.

There are checks which serve to delay, perhaps fortunately, the too precipitate eradication of errors engrafted upon truth. Some of them originate in the fears of those who stand in the advanced ranks of thought and investigation, as to the propriety of disturbing, without careful preparation, the settled convictions of the people on points which they have been heretofore taught were essential—preferring rather to let the truth develop itself in the general education and cultivation of the growing generations.

Other checks come in the shape of partial sectarian education which, clinging to old dogmas, sends the youth from school to learn in after life, if not blinded by bigotry, the lesson which had been withheld or garbled by his teacher.

I repeat there is nothing in the developments of science nor in the advanced stages of thought, where truth forms the basis and the structure, which in any way interferes with the relations of man to his Creator or to his fellow-man. On the contrary, as we progress, eradicating errors of the past and grasping and utilizing the wonderful scientific revelations granted to us in the present, we come nearer to Him and with each step comes a better appreciation of His glory and power, and to the well-balanced mind a fuller and more perfect trust. A desire to properly fulfill our duty here and a willingness to resign ourselves to His will in the future.

Why I have penned these thoughts for the BISTOURY I do not know. I seem to have been *moved* to it—and perhaps they may not be so entirely out of place in a journal whose mission is to aid in relieving the diseases of men. Mental as well as physical sufferings are equally within the province of the physician, for either is a sure attendant upon the other, and possibly this may reach some who, failing in the wreck of misplaced hopes, "seeing, may take heart again."

